# Bluues_cplx: Electrostatics at Protein–Protein and Protein–Ligand Interfaces

**DOI:** 10.3390/molecules30010159

**Published:** 2025-01-03

**Authors:** Miguel Angel Soler, Rayyan Bassem Adel Yakout, Ozge Ozkilinc, Gennaro Esposito, Walter Rocchia, Christian Klein, Federico Fogolari

**Affiliations:** 1 Dipartimento di Scienze Matematiche, Informatiche e Fisiche (DMIF), University of Udine, 33100 Udine, Italy; miguelangel.solerbastida@uniud.it (M.A.S.); ozkilinc.ozge@spes.uniud.it (O.O.); 2 Institute of Biotechnology, IMC Krems University of Applied Sciences, 3500 Krems an der Donau, Austria; 21imc10505@imc.ac.at (R.B.A.Y.); christian.klein@imc.ac.at (C.K.); 3 Science and Math Division, New York University at Abu Dhabi, Abu Dhabi P.O. Box 129188, United Arab Emirates; rino.esposito@nyu.edu; 4 Computational MOdelling of NanosCalE and BioPhysical SysTems CONCEPT Lab, Istituto Italiano di Tecnologia (IIT), 16152 Genoa, Italy; walter.rocchia@iit.it

**Keywords:** bluues, electrostatics, protein, free energy, software, ligand, complex

## Abstract

(1) Background: Electrostatics plays a capital role in protein–protein and protein–ligand interactions. Implicit solvent models are widely used to describe electrostatics and complementarity at interfaces. Electrostatic complementarity at the interface is not trivial, involving surface potentials rather than the charges of surfacial contacting atoms. (2) Results: The program bluues_cplx, here used in conjunction with the software NanoShaper to compute molecular surfaces, has been used to compute the electrostatic properties of 756 protein–protein and 189 protein–ligand complexes along with the corresponding isolated molecules. (3) Methods: The software we make available here uses Generalized Born (GB) radii, computed by a molecular surface integral, to output several descriptors of electrostatics at protein (and in general, molecular) interfaces. We illustrate the usage of the software by analyzing a dataset of protein–protein and protein–ligand complexes, thus extending and refining previous analyses of electrostatic complementarity at protein interfaces. (4) Conclusions: The complete analysis of a molecular complex is performed in tens of seconds on a PC, and the results include the list of surfacial contacting atoms, their charges and Pearson correlation coefficient, the list of contacting surface points with the electrostatic potential (computed for the isolated molecules) and Pearson correlation coefficient, the electrostatic and hydrophobic free energy with different contributions for the isolated molecules, their complex and the difference for all terms. The software is readily usable for any molecular complex in solution.

## 1. Introduction

Most biological functions rely on highly specific and diverse biomolecular recognition [1]. The shape and electrostatic complementarity of the interacting molecules in the complex, whether (statistically) existing before, or induced by complexation, have been recognized very early as determinants of recognition of substrates by enzymes. The concept of lock and key introduced by Fischer [2] was later expanded with the introduction of induced-fit [3] and conformational selection [4] mechanisms, by Koshland and Nussinov and collaborators, respectively. As soon as the first complexes structures had been solved, the idea of shape and polarity complementarity was verified also for protein–protein complexes [5]. According to Chothia and Janin, the driving force for binding was provided by the burial of a hydrophobic surface upon complexation with polarity playing the role of fine tuning the interaction. Polarity complementarity was checked by considering hydrogen bonding at the interface.

In the early 1990s, the availability of programs that solve the Poisson–Boltzmann equation in solution, like DelPhi [6] and UHBD [7], made it possible to consider the electrostatic potential rather than just atomic partial charges or Coulomb potential. The work of Colman and coworkers [8] studied 12 protein–protein complexes and the role of electrostatic complementarity as defined by both charge and potential patterns at interfaces. Their study showed that rather unexpectedly, charge–charge complementarity at neighboring atoms was insignificant with correlation coefficients close to zero.

They also computed (i) the electrostatic potential of the fully solvated proteins (as excised from the complex) at their own surface and at the points where the surface of the other interacting protein would be located in the complex; and (ii) the electrostatic potential of the partially solvated proteins (the complex) at their own surface and at the surface of the other interacting protein.

For each surface point, the self potential and the potential due the other protein, calculated as said above, were paired, and in this case, both the Pearson and Spearman correlation coefficients were significant both for the fully and partially solvated systems. For the fully solvated systems, the Pearson correlation coefficient was remarkably smaller than the Spearman correlation coefficient, but overall, the study showed that electrostatic complementarity is best represented by surface potential rather than by the charges of surface exposed atoms.

Other studies using the Poisson–Boltzmann focused on the calculated electrostatic free energy of binding (assuming mostly rigid docking). Although implicit solvent calculations performed on rigid interacting molecules can be at most qualitative, it appeared that electrostatic contributions are mostly non-favorable to binding, because the computed electrostatic free energy of binding is found to be inversely correlated with the area of the buried surface [9]. Moreover, charge dispersion as the result of networks of hydrogen bonds and ionic pairs, often exposed to the solvent in the complex, suggests that the desolvation energy is strongly opposing binding. Of the many studies addressing the role of electrostatics in protein–protein interactions in the frame of implicit solvent calculations, the study of Alexov and collaborators [10] is particularly important because it tested in a systematic way the influence of continuum models’ parameters on the computed properties. Under all reasonable choices for the dielectric constant and surfacing probe radius, the electrostatic free energy of binding was unfavorable for homo-dimers and was favorable for heterodimers only for a high dielectric constant and taking the van der Waals surface as the solute–solvent interface, as suggested by Dong and Zhou [11], who had shown that this choice was correlating better with experimental free energies for mutants.

More recently, Grassmann et al. [12] related electrostatic and shape complementarity to binding affinity, showing that higher binding affinity is associated with high values of shape complementarity, whereas higher electrostatic complementarity characterizes lower binding affinity complexes. Their analyses are based on the idea of expanding the surface potential projected on a disk, best fitting the interface, using 2D Zernike polynomials. The authors made their software publicly available. The latter provides very efficiently a measure of shape and electrostatic complementarity.

For protein–ligand complexes, the electrostatic complementarity at interfaces was quantitatively studied by Chau and Dean [13], mapping the potential due to the host and the ligand at the ligand surface. The potential was computed using a fixed or a distance-dependent dielectric constant with similar results. The conclusion was that the complementarity (as measured by Pearson correlation coefficient) of charges was very small, whereas the potentials due to the host and ligand were strongly anticorrelated.

The importance of electrostatic complementarity in protein–ligand interactions has been demonstrated by showing that its optimization leads to more favorable binding free energies [14,15]. Zhao et al. further modified the definition of electrostatic complementarity and showed that it was even more correlated with ligand activity [16].

Kuzic and Nielsen [17] reviewed the application of continuum models based on the Poisson–Boltzmann equation [18] and Generalized Born radii [19,20] approaches to protein–ligand thermodynamics.

Notwithstanding the importance of continuum models and the availability of software for the computation of electrostatic effects, there is currently no software available, based on such models, which performs a complete analysis of electrostatic binding free energy, buried surfaces, atom–atom contacts and surface–surface contacts with potential pairs, thus providing the analysis of electrostatic complementarity and related quantities.

Here, we describe and make available a software, bluues_cplx (URL https://github.com/federico-fogolari/bluues_cplx accessed on 30 December 2024), which uses Generalized Born (GB) radii [19,20], computed by a molecular surface integral, to output several descriptors of electrostatics at protein (and in general, molecular) interfaces. Similar information is not available in the output of programs like Bluues2 (URL https://github.com/federico-fogolari/bluues2 (accessed on 30 December 2024))  [21,22] working with a single molecular entity. In order to obtain the same information by Bluues2 or any other software, a pipeline of independent calculations should be performed and the output of each calculation parsed and used in slow shell scripts. Solvent excluded surfaces are efficiently generated here using the software NanoShaper v. 0.7.8 [23]. The program has been tested on 756 protein–protein and 189 protein–ligand complexes. The results are consistent with and extend previous investigations. The complete analysis of a molecular complex is performed in tens of seconds on a PC, and the results include the list of surfacial contacting atoms, their charges and Pearson correlation coefficient, the list of contacting surface points with the electrostatic potential (computed for the isolated molecules) and Pearson correlation coefficient, the electrostatic and hydrophobic free energy with different contributions for the isolated molecules, their complex and the difference for all terms.

Bluues_cplx is readily usable for any molecular complex in solution.

## 2. Results and Discussion

### 2.1. Test on Protein–Protein Complexes

**Figure 1:**
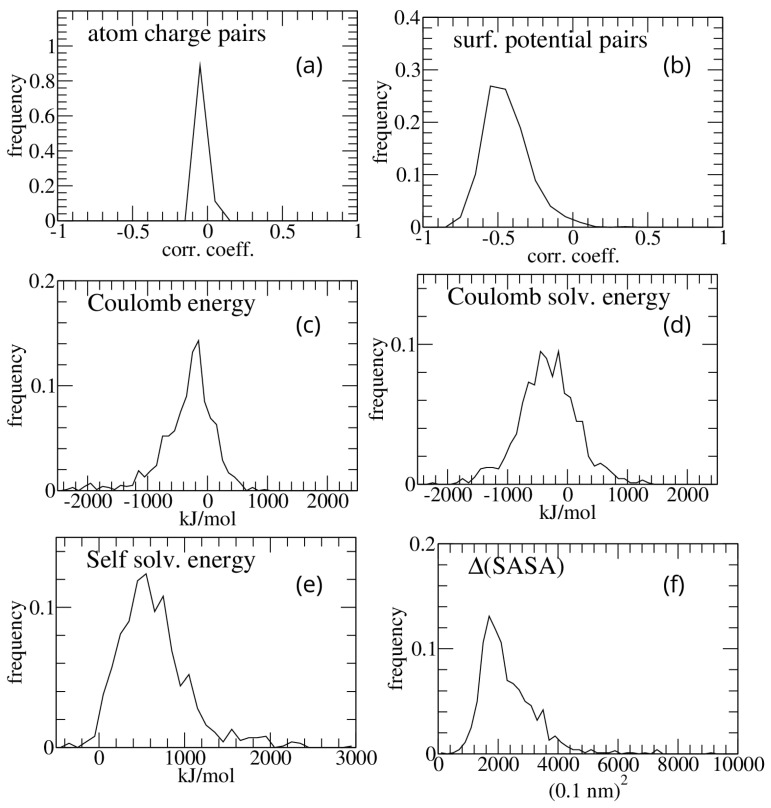
Frequency distributions of the results obtained on the PDBbind protein–protein complex dataset. (**a**) Atomic charge pairs correlation coefficients; (**b**) surface potential pairs correlation coefficients; (**c**) interaction Coulomb energy; (**d**) difference (bound minus isolated molecules) in GB solvation energy of Coulombic interactions; (**e**) difference (bound minus isolated molecules) in self-solvation GB energy; (**f**) difference (bound minus isolated molecules) in solvent accessible surface area.

Bluues_cplx was first applied on the PDBbind dataset. Several quantities were taken as possible measures of electrostatic complementarity. All pairs of atomic charges (each of which originates from one atom of the two interacting molecules) at a distance less than the sum of their van der Waals radii plus 1 Å were selected, and their correlation coefficient was computed for each complex. The distribution of such correlation coefficients is shown in Figure 1a.

Consistent with previous results in the literature [8,12], the atomic charge mean correlation is negative but close to zero (−0.016 ± 0.014), which is a result that parallels the negative but very wide distribution of Coulomb interaction energy (−326.7 ± 446.6) (Figure 1c).

In contrast to the correlation of atomic charges of the interacting molecules, which is close to zero, the electrostatic potentials on solvent-excluded surface points within 0.1 nm of each other show a much more negative correlation (−0.44 ± 0.15). The corresponding distribution over the analyzed complexes is shown in Figure 1b. Other components of the interaction free energy are displayed in Figure 1d–f.

**Figure 2:**
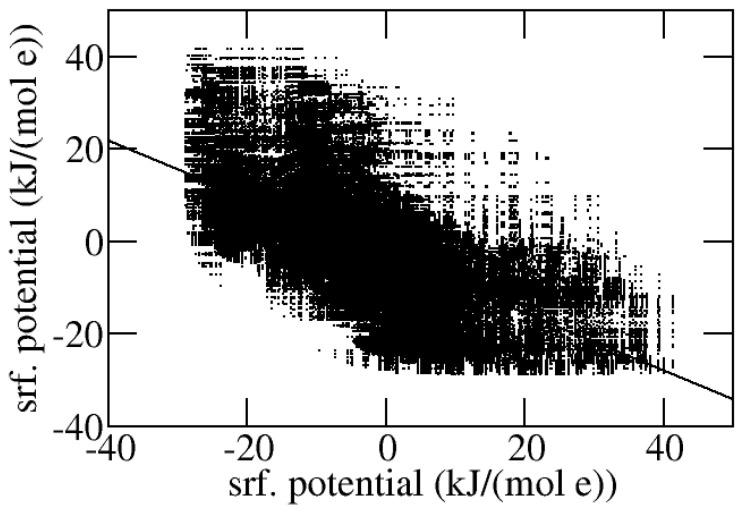
Ribonuclease SA/inhibitor barstar complex. For each pair of surface points in close contact (within 1 Å), the respective potentials are shown as a point. Each potential pair is displayed twice by swapping coordinates. The linear regression line (slope = −0.62, intercept = −3.0 kJ/(mol e)), where *e* is the absolute charge of the electron, is also displayed.

The program ouputs a list of surface potential pairs (in the Methods example, the file out.srf_pot_pair) referring to surface elements on the two different interacting molecular entities which are closer than the cutoff and the atoms contacting the solvent molecules at those surface elements in the free molecular entities. An example of the latter pairs as output for the complex of Ribonuclease SA and the inhibitor barstar [24] (pdb id. 1ay7) is plotted in Figure 2, together with the linear regression line. For this complex, which displays a potential pair correlation coefficient equal to −0.62, the interacting surfaces display strong complementarity.

**Figure 3:**
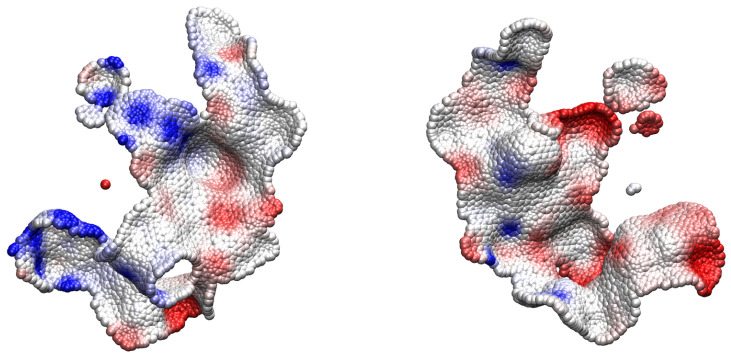
Ribonuclease SA/inhibitor barstar complex. Contacting surface points on guanyl-specific ribonuclease SA (**left**) and barstar barnase inhibitor (**right**) are shown as spheres. The potential is shown in color code (saturated blue: 25.0 (kJ/(mol e)), saturated red: −25.0 (kJ/(mol e))).

The program outputs also the coordinates of the surface points in close contact, which is in pdb format together with the potential in kJ/(mol e) in the temperature factor field. The latter pdb files can be displayed using one of the standard molecular visualization softwares like VMD (URL https://www.ks.uiuc.edu/Research/vmd/, accessed on 30 December 2024) [25]. As an example, the two interacting surfaces are displayed with the potential to color code in Figure 3, where the anticorrelation of the potentials is apparent.

The correlation between the calculated energies (whose distribution is reported in Figure 1c–f) and the binding free energies reported in the PDBbind set for 756 complexes is rather poor. The largest correlation coefficients are found with the buried solvent accessible surface area (Pearson correlation coefficient 0.14) and with the sum of products of the potentials in contacting surface points (Pearson correlation coefficient 0.13).

In order to compare our results with previous studies, we considered the fraction of contacting surface points having potential with the same sign, which were analogous to “concordant” pixels in the study by Grassmann et al. [12]. The authors of that study took all pixels pairs, as computed from true complexes, and considered all pairs obtained by randomly resampling the surface points. The fraction of concordant pixels was used as a predictor of true versus decoy complexes. In a Receiver Operator Characteristic (ROC) analysis, the Area Under Curve (AUC) was ranging between 0.59 and 0.62 for the complexes formed at 5.5 < pH < 7.5. We performed the same analysis on the PDBbind protein dimers dataset, finding 0.418 as the best discriminative value, with an AUC equal to 0.761. The better performance could be expected because of the less informative 2D-Zernike polynomial representation of contacting surfaces, which has, however, its own specific advantages [12]. It is worth noting that the Pearson correlation of the potential values of contacting surface points is almost a perfect discriminator with an AUC equal to 0.988.

Unfortunately, real decoys, like those obtained from the CAPRI experiment [26], display much larger electrostatic complementarity than that obtained by randomly resampling surface electrostatic potentials.

**Figure 4:**
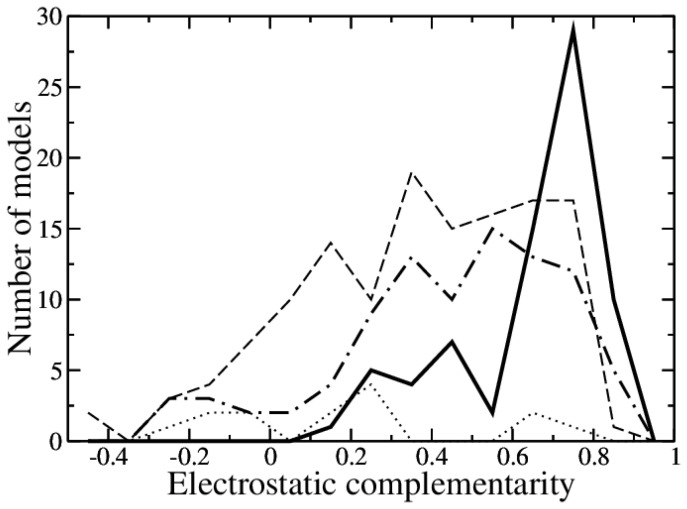
Histogram of electrostatic complementarity distributions for high-quality (coninuous line), medium-quality (dot-dashed line), acceptable (dotted line) and incorrect (dashed line) models. The range −0.5 to 1 is divided in 0.1 bins.

To illustrate the latter point and to provide a realistic application case, we consider the set of models deposited by predictors for evaluation for the target T153 in the CAPRI experiment, i.e., the most recent available target which also contains high-quality predictions. The set of 313 predictions has been rescored according to electrostatic complementarity as defined in the Materials and Methods section. The set contains 135 incorrect, 14 acceptable, 91 medium and 73 high-quality predictive models. Although it is true that in general, high-quality models display better electrostatic complementarity than incorrect models, it is also true that some predictors clearly improved electrostatic complementarity compared to others, which was possibly at the expenses of other non-optimized features. Thus, the two models with the largest electrostatic complementarity are indeed high-quality models, and among the ten models with the largest electrostatic complementarity, five are high quality and four are medium quality, but also an incorrect model is found. It turns out that all the models by the same predictor display large electrostatic complementarity. The distribution of electrostatic complementarity for high-quality, medium-quality, acceptable and incorrect models is shown in Figure 4, where the extensive overlap of the distributions for the models of the four categories is apparent. The usefulness of electrostatic complementarity as a criterion to rescore and filter predictive models is, however, demonstrated by the fact that out of the best ten percent rescored models, half of them are high-quality models.

### 2.2. Test on Protein–Ligand Complexes

Bluues_cplx was applied on the protein–ligand v2013-core set of the PDBbind dataset. Overall, 189 out of 195 complexes could be processed automatically, and the analyses were performed on this set.

We considered exactly the same quantities as for the protein–protein complex dataset.

**Figure 5:**
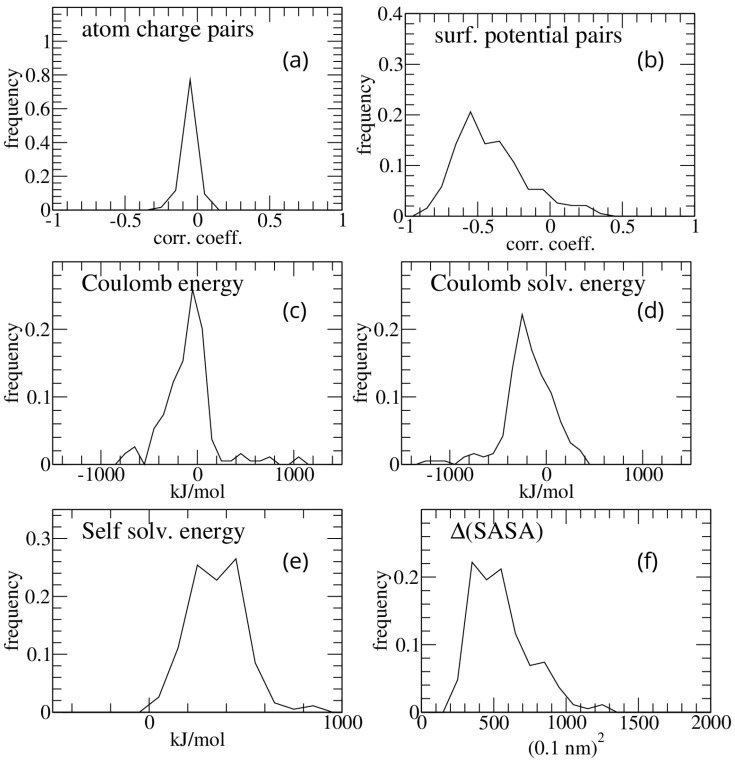
Frequency distributions of the results obtained on the PDBbind v2013-core protein–ligand dataset. (**a**) Atomic charge pairs correlation coefficients; (**b**) surface potential pairs correlation coefficients; (**c**) interaction Coulomb energy; (**d**) difference (bound minus isolated molecules) in GB solvation energy of Coulombic interactions; (**e**) difference (bound minus isolated molecules) in self-solvation GB energy; (**f**) difference (bound minus isolated molecules) in solvent-accessible surface area.

The distribution of the correlation coefficients obtained for each complex for the charges of the contacting atoms is shown in Figure 5a.

Similar to what we saw for protein–protein interfaces, the correlation is negative but close to zero (−0.05 ± 0.05), which is a result that parallels the negative but very wide distribution of Coulomb interaction energy (−93.4 ± 264.5); see Figure 5c.

The correlation coefficients of the potential of pairs of solvent-excluded surface points (on different molecules) within 0.1 nm of each other are also in this case negative: −0.40 ± 0.24 (Figure 5b).

Due to smaller interfaces (the mean buried solvent-accessible surface area is 549 +/− 203 Å^2^ for protein–ligand interfaces versus 2330 +/− 969 Å^2^ for protein–protein interfaces), the distributions shown in Figure 5 display larger dispersions compared to the protein–protein interfaces.

**Figure 6:**
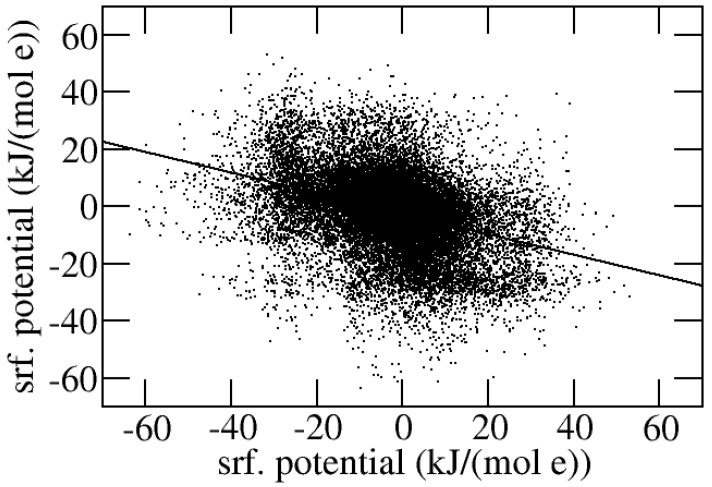
A set of 17,455 representative pair potentials referring to surface points in close contact (within 1 Å), taken from all the protein–ligand interfaces, are shown as points. Each potential pair is displayed twice by swapping coordinates. The linear regression line (slope = −0.36, intercept = −2.4 kJ/(mol e)) is also displayed.

The surface potential pairs referring to the surface elements of the protein and the ligand which are closer than 1 Å in the complex are plotted in Figure 6 together with the linear regression line. For readability, only a pair in 1000 is shown. The regression line has slope −0.36 and intercept −2.4 kJ/(mol e).

Also, here, the correlation between the calculated energies (and their components whose disributions are reported in Figure 5d–f) and the binding free energies reported in the PDBbind set is very poor. In the protein–ligand complexes, only the buried solvent-accessible surface area displays a very large Pearson correlation coefficient (−0.56) with the binding free energy, confirming that surface burial provides the main driving force also for drug binding.

In order to compare our results wih previous studies, and to provide a realistic application scenario, we considered the datasets used by Bauer et al. [14] in their study. The datasets are thoroughly described in the referenced paper. The authors were able to show that electrostatic complementarity is significantly correlated with the experimental binding energies. We assigned ligand charges and radii, using the Gasteiger and Marsili method [27] as implemented in the software OpenBabel (URL https://openbabel.org/, accessed on 30 December 2024)  [28], and for the protein, we used the forcefield AMBER ff14SB [29] as implemented in ChimeraX 1.9 [30]. The results are reported in Table 1 where similar trends are apparent. It is worth mentioning that the authors of the reference study used the proprietary eXtended Electron Distribution (XED) forcefield [31], which has a much more accurate representation of atomic and electronic charges compared to the atom-centered charges used here. We attribute the difference in performance reported in Table 1 to the quality of the forcefield employed rather than to the definition of the electrostatic complementarity, which is very similar in our study and their study.

## 3. Methods

### 3.1. Generalized Born Methodology Implementation

Electrostatics in non-homogeneous media is described by the Poisson equation and, in the presence of salts, under specific reasonable assumptions, by the Poisson–Boltzmann equation [18].

An alternative approach to treat electrostatics of biomolecules in solution is based on the Generalized Born (GB) approach to compute the electrostatic interaction and solvation energies, which has been described in excellent reviews [19,20].

The advantage of the GB approach is that it allows the dissection of contributions to the potential and therefore to electrostatic free energies. Pairwise interactions, where the rest of the molecule enters implicitly the Generalized Born radius, can also be computed, as discussed hereafter.

The methodology has been implemented here essentially as in the software Bluues2  [22]. Here, however, the focus is on interactions and interfaces rather that single molecular entity properties. All the information on interfaces and interaction energies and potentials is obtained in the output of the program, whereas it would require different runs of different programs, parsing outputs and combining them by likely slow scripts if the program Bluues2 was to be used.

In bluues_cplx, the computation of Generalized Born radii is performed by a discretized surface integral [21,32]. First, based on atomic radii, the solvent-excluded surface is computed calling the program NanoShaper [23] or alternatively MSMS [33]. The surface is then used to compute the Generalized Born radius as previously described [21,32].

Once Generalized Born radii (which depend on all atoms within a chosen cutoff) are computed, the calculation of solvation free energies is performed analytically, and different free energy components are listed in the output. The analytical calculation entails single and pairwise atomic terms, and therefore, it is possible to assign to each atom an electrostatic free energy contribution, although Generalized Born radii, which enter all calculations, are inherently multibody quantities.

In practice, the software takes as input a pqr file, i.e., essentially a PDB file with charge and radius following coordinates for each ATOM record. The conversion from PDB to PQR format can be performed using the server pdb2pqr (available at URL: https://server.poissonboltzmann.org/pdb2pqr, accessed on 30 December 2024) [34,35] or the stand-alone code [36]. For general compounds, the software OpenBabel offers also conversion from many input formats to pqr with options to calculate partial charges [28]. It is important at this step to keep the chain of each atom as in the original PDB file, e.g., with the option “--keep-chain” with pdb2pqr or by ticking the corresponding option in the web server window, because bluues_cplx uses the chain field to define interacting molecules.

Together with the input file, the program reads on the command line two strings (possibly containing only a single character) whose characters define the chains of the interacting molecules. Additional options are listed by running the program without arguments.

A command example is the following (once bluues_cplx is in the command path):

bluues_cplx cplx.pqr out A BC -ns aux

with two mandatory arguments (in this case “cplx.pqr” “out” “A” and “BC”) followed by additional opions (in this case “-ns” followed by specification “aux”).

The program reads the pqr file cplx.pqr, considers chain A as interacting entity 1 and chains B and C together as interacting entity 2. All output results will have as basename “out”; i.e., for example, the output energy difference will be in the file out_diff.nrg, the surface potential pairs in the file out.srf_pot_pair, etc. The program will use NanoShaper to generate the SES with input and output files basename “aux”.

Three different calculations are performed: one for the complex and one for each of the two isolated interacting molecules. For each calculation, Generalized Born radii are first calculated, and then, using GB radii, the program computes the surface potential at each surface point, the total energy and the atomic contributions with the caveat reported above about the multibody character of Generalized Born radii.

Since GB radii are computed by integration over the surface which is generated by the program NanoShaper [23] or alternatively MSMS [33], the latter programs must be installed and be available to the user as “NanoShaper”, as detailed in the next subsection, or “msms”, respectively.

A comparison between the SES calculated by NanoShaper and MSMS on systems of different size, as well as the impact of an accurate SES calculation on the subsequent solution of the Poisson–Boltzmann equation, are provided in Refs. [23,37].

Bluues_cplx prepares a file with coordinates and radii with extension “.xyzr”, and, if NanoShaper is used, an input file for NanoShaper based on the input structure. In all tests performed, the generated parameters were appropriate, and the program was completed successfully.

Surface points are extracted from the triangulated surface, and each surface point is at the center of a surface patch associated with an area and a normal vector.

Based on a short-range cutoff used to define contacts (the default is 1 Å), all contacts between surface points are listed together with their potential and the solvent excluding atom.

With the same cutoff, all atoms whose van der Waals surfaces are closer than the short-range cutoff are listed with their charges.

Finally, differences in free energy between complex and isolated molecules for all atoms are listed. The output consists of triplets of files (one for the complex and two for the interacting molecular entities, which are generically referred to as mol1 and mol2, even if each of them may consist of more than one molecule), e.g., for the output basename “out” and for the NanoShaper input/output basename “aux”:

- out_int.pqr, out_int_mol_1.pqr, and out_int_mol_2.pqr pqr files with the atoms within a chosen cutoff from the interface;
- out.nrg, out_mol_1.nrg, out_mol_2.nrg, and out_diff.nrg free energy files with total Coulomb and solvation free energy. The Coulomb term is just the Coulomb interaction between fixed charges in the molecule. The solvation term is further split in a Born desolvation free energy for each fixed charge, in a Coulombic interaction solvation free energy (for each pair of fixed charges), and a solvation energy proportional to the solvent accessible surface through a surface tension coefficient, here taken as constant independent of the atom type. The contribution of each atom to the total energy is listed for the atoms within the chosen cutoff from the interface (except for the GB radii dependence on all other atoms). The last file contains the difference in all the above free energy terms;
- out.gbr, out_mol_1.gbr, and out_mol_2.gbr gbr files with the GB radii and out.pqg, out_mol_1.pqg, and out_mol_2.pqg pqg files which are pqr files where the van der Waals radius is replaced by the GB radius.

Four output files contain information about electrostatic complementarity:

- out_mol_1.srf and out_mol_2.srf containing the portions of the two molecular surfaces which are in close contact in the complex, outputted in pdb format with the position of the surface point in coordinate fields and its potential in the temperature factor field;
- out.crg_pair all pairs of atoms, belonging each to a different molecular entity, whose van der Waals surfaces are within a short cutoff (default 1 Å) are listed with their charge, and at the end of the file, the Pearson correlation coefficient and the sum of charge-charge products;
- out.srf_pot_pair listing all pairs of solvent-exposed surface points, belonging each to a different molecular entity, which are within a short cutoff (default 1 Å), with the computed surface potential (for details, see references describing the program Bluues2 [21,22]), and at the end of the file, the Pearson correlation coefficient and the sum of potential products. The latter sum depends on the extent of the interface but also on the density of surface points. Note that the surface of each molecular entity is computed in the absence of the other one.

Finally, there is a summary file:

- out.summary, which contains the most relevant quantities from the analysis. If the flag -lite is used, this is the only file outputted by the program.

### 3.2. Electrostatic Complementarity

We define the electrostatic complementarity of a complex in the following way. First, we separate the two interacting molecules (or groups of molecules), and we calculate the solvent excluded surfaces and the electrostatic potential at each surface point. Then, for all pairs of surface points which are closer than a cutoff in the complex, the pair of surface potential values is listed.

Since the surface potential is not typically generated using the Generalized Born approach, we remark that the potential at the surface is generated placing a test charge at the surface point. The radius of the charge was optimized in order to best reproduce the results obtained with the Poisson–Boltzmann equation [21].

The Pearson correlation (with sign changed) computed for all the pairs of surface potential values is used here as a measure of electrostatic complementarity.

### 3.3. Solvent-Excluded Surface (SES) Calculation

Whereas solvent-accessible surface (SAS) is computed autonomously by bluues_cplx as previously described [21], SES must be computed by an external program.

Compared to the parent software Bluues2 [22], which could use the program MSMS [33] for the calculation of the SES, bluues_cplx can use also the NanoShaper tool [23]. In the present work, we used the latter for all calculations.

NanoShaper and/or MSMS must be independently installed on the system and be available as system commands “NanoShaper” and “msms”, respectively. Executables of the two programs are available at URLs https://gitlab.iit.it/SDecherchi/nanoshaper/-/raw/Release-0.7.8/pkg_nanoshaper_0.7.8.tar.gz (accessed on 30 December 2024) and https://ccsb.scripps.edu/msms/download/933/msms_i86_64Linux2_2.6.1.tar.gz (accessed on 30 December 2024), respectively. Instructions on how to install the programs are reported in the documentation of the same programs and also in the file READ.ME in the bluues_cplx package.

We provide hereafter a brief description of NanoShaper which is more recent than the well-known program MSMS [33].

NanoShaper is a software for building and analyzing the molecular surface. It can use three different definitions: SES (or Connolly), Skin and Blobby Gaussian surfaces. In the case of the SES, it builds a mathematical model where the space is divided in Voronoi cells and the equation of the surface patch for each cell is calculated. Again, in this case, the patches can be either portions of spheres or of tori. Then, coordinate-axis-aligned rays are cast with a user-defined resolution and the corresponding intersections between the rays and the surface patches are calculated with high accuracy. This information is used to triangulate the surface as well as to color a grid for successive PDE resolution. The mathematical model provides both the surface component facing the external solvent and the components related to internal cavities that can be kept or removed, depending on the application [38].

In the output of NanoShaper, there are the points of the surface triangulation together with the corresponding normal, the surface area of the different components of the surface, the volume of the entire molecule, the volume of each internal closed cavity, and the list of the atoms that locally contribute to the surface.

Bluues_cplx generates an input file for NanoShaper with parameters automatically determined by the size of the input molecular structures. In particular, most parameters are fixed or determined by the options (like the probe radius or filenames), whereas Grid_scale and Grid_perfil are set heuristically to $\frac{1}{\sqrt{1.6\times \mathrm{surface}\;\mathrm{element}\;\mathrm{area}}}$ and $\frac{90}{90+\mathrm{number}\;\mathrm{of}\;\mathrm{atoms}}\times 100$, respectively. Such a procedure was found to work on all test input structures. The choices Grid_scale 2.0 and Grid_perfil 95 should, however, work for all cases. The user can use an existing input parameter file, thus bypassing its generation, using the flag -rpns.

### 3.4. Test Datasets

As a test set for protein–protein complexes, we used the dataset from PDBbind (v. 2020) which entails 2852 protein complexes [39] with binding data. From this set, only bimolecular complexes were considered, and further, for all complexes with at least one chain sharing more than 90% identity with some chain of another complex, only one representative was chosen. The latter analysis was performed using the software Cd-hit [40]. At the end of the procedure, 756 complexes were considered. The choice of considering only bimolecular complexes was taken just to avoid arbitrary choices or time-demanding exhaustive testing of all possible partition of molecular chains in two interacting entities.

As a test set for protein–ligand complexes, we used the dataset v2013-core from PDBbind (v. 2020), which entails complexes and binding data for 65 proteins with three different ligands each, totaling 195 complexes [39].

## 4. Conclusions

In this work, we described a software that is able to compute different electrostatic and surface properties which may be relevant for binding. The output of the program includes electrostatic free energy of binding, pairs of contacting atoms and their charges, pairs of contacting surface points and their potential and Pearson correlation coefficients for charge and potential pairs.

These quantities may be helpful in rationalizing binding energies and in designing better binders.

The tests performed on protein–protein and protein–ligand complexes show that indeed, the electrostatic complementarity as measured by the Pearson correlation coefficient of the potential pairs at contacting surface is largely negative.

On the other hand, the correlation of the Pearson correlation coefficients and the computed binding free energies, and their contributions, with the experimental binding free energies is rather poor. The latter conclusion is not contradicting the importance of electrostatic complementarity in determining the free energy of binding, which has been repeatedly demonstrated in previous studies [14,15]. Typically, such studies compare series of similar compounds so that additional contributions, which may be largely different for unrelated compounds and targets, cancel out in comparison, which is not the case here where different complexes are pooled together.

In the tests performed on the dataset used by Bauer et al. [14], which indeed contains a series of ligands for the same target, a good correlation between drug activiy/binding free energy and electrostatic complementarity is found.

This is consistent with the idea that the driving force for complex formation is largely hydrophobic, whereas electrostatic interactions are essential in tuning and modulating molecular interactions[14,15,41].

There are several limitations of the approach used here to compute the thermodynamic properties of molecular complexes, which is worth mentioning here:

(i) Electrostatic free energies and most of their contributions are extremely large and sensitive to minor molecular details. The model used to assign charges and radii has a profound influence on the result;

(ii) Molecules are treated rigidly. Sometimes, small changes in coordinates can change dramatically solvent accessibility, which in turn leads to large changes in solvation free energy. Fluctuations in structure and therefore in electrostatic properties are not considered. Note that binding free energies are typically one or more orders of magnitude smaller than the contributions from which they are computed. Even limited relative inaccuracies in each term can lead to large errors in their differences;

(iii) For the above reasons, the best results can be obtained only when comparing very similar complexes due to the expected cancellation of inaccuracies. Such an example is provided here by the analyses of the datasets of Bauer et al. [14].

Notwithstanding the limitations mentioned above, the software can be used for the characterization of the electrostatic potential at interacting surfaces, to gain qualitative information on driving forces of binding, and to predict the effect of changes (e.g., mutations in protein or DNA or chemical group substitutions in ligands) on binding thermodynamics.

In summary, our sofware will help the proper characterization of electrostatics, within the implicit solvent framework provided by the Generalized Born model, for molecular complexes in solutions.

## Figures and Tables

**Table 1:** Correlation of potential–potential complementarity with bioactivity for the ligand–protein datasets of Bauer et al. [14].

Dataset (PDB Id.)	Number of Ligands	Bluues_cplx	Bauer et al., 2019 [14]
mGLU5 (5CGC)	11	0.39	0.67
mGLU5 (5CGD)	10	0.57	0.52
XIAP (5C7D)	11	0.52	0.65
XIAP (5C7A)	11	0.59	0.60
PIM1 (5VUC)	6	0.67	0.84
Mcl-1 (6B4L, all)	40	0.03	0.18
Mcl-1 (6B4L, Chlorine scan)	17	0.33	0.36
MEK1 (3DY7)	4	0.72	0.94
Imatinib-kinase (various)	9	0.30	0.52
biotin-SA (3RY2)	4	0.89	0.86

## Data Availability

The list of pdb IDs of all protein–protein complexes and protein–ligand complexes used for testing in this work are reported as a Supplementary Material file with the detail of how charges and radii have been assigned. The software bluues_cplx with instructions for compilation and usage is provided as a zip archive file in Supplementary Material and is available at URL: https://github.com/federico-fogolari/bluues_cplx (accessed on 30 December 2024).

## Supplementary Materials

The following supporting information can be downloaded at: https://www.mdpi.com/article/10.3390/molecules30010159/s1.
