# Supplementary material for: Bluues_cplx: Electrostatics at Protein–Protein and Protein–Ligand Interfaces
Source: Molecules. 2025 Jan 3;30(1):159. doi: 10.3390/molecules30010159 (PMC11722155; doi:10.3390/molecules30010159)
Supplement: Supplementary file 1 [file molecules-30-00159-s001.zip › list_pdb_ids.pdf]

List of the pdb ids of protein-protein complexes and protein-ligand complexes

Protein charges and radii assigned using the software PDB2PQR 3.0 (AMBER forcefield)
Ligand charges and radii obtained from mol2 files in the PDBBIND database (Liu, Z.; Su, M.; Han, L.; Liu, J.; Yang, Q.; Li, Y.; Wang, R. Forging the basis for developing protein#ligand interaction scoring functions. Accounts of chemical research 2017, 50, 302-309).

List of pdb ids of protein-protein complexes:

```
1a22 1acb 1an1 1avx 1axi 1ay7 1buh 1bvn 1c9p 1clv 1d6r 1dhk
1djs 1dp5 1dpj 1e96 1ees 1eja 1emv 1es0 1f34 1f3v 1f5r 1f7z
1fle 1fmo 1fy8 1g0v 1g5j 1g6v 1g9i 1gl0 1gla 1grn 1gua 1h0t
1h1v 1h59 1he8 1j2j 1j7d 1j7v 1jgn 1jh4 1jiw 1jsp 1jtd 1kac
1kbh 1ktz 1kxp 1l4d 1l4z 1l8c 1ldt 1lp1 1lw6 1lx5 1lzw 1m10
1m1e 1m5n 1mah 1mcv 1mzw 1nw9 1o9a 1oc0 1op9 1oph 1otr 1oyh
1ozs 1p69 1p6a 1p9d 1pd7 1pjm 1pjn 1ppe 1q0w 1q5w 1q68 1q69
1r0r 1r8u 1ri8 1rjc 1rkc 1rke 1ry7 1sb0 1shy 1smf 1sq0 1syq
1t01 1t0p 1t44 1t5z 1t63 1t6b 1ta3 1taw 1tba 1tdq 1te1 1tlh
1tm1 1tm3 1tm4 1tm5 1tm7 1to1 1u0i 1u0s 1u5s 1uel 1ugh 1vet
1veu 1vg0 1vrk 1wa7 1wa8 1wlp 1wq1 1wqj 1wr1 1wrd 1xdt 1xg2
1xj7 1xr0 1xt9 1y1k 1y33 1y34 1y3b 1y3c 1y3d 1y48 1y4a 1y4d
1y6k 1y6m 1y6n 1y8n 1yc0 1ydi 1yrt 1yru 1yvb 1yx5 1yx6 1z92
1zgu 1zhi 1zjd 1zli 1zsg 1zv5 1zvh 1zvy 2a24 2a78 2a7u 2a9k
2aq2 2arp 2b0z 2b12 2b42 2b7c 2b87 2c0l 2c1m 2cpk 2den 2djy
2dsp 2dx5 2f31 2f4m 2few 2fju 2ftl 2ftm 2fuh 2fyl 2g2u 2g2w
2g81 2gng 2gww 2hd5 2hev 2hle 2hqw 2hrk 2hsq 2hth 2j12 2jby
2jgz 2jod 2jt4 2jti 2ju0 2jy6 2k2s 2k2u 2k3s 2k42 2k5b 2k6d
2k79 2k7a 2k8b 2k8c 2k8f 2ka4 2ka6 2kbw 2kc8 2kgx 2khs 2kj4
2knb 2kqs 2kri 2kt5 2ktf 2kvq 2kwi 2kwj 2kwk 2kwo 2kwu 2kwv
2kxh 2kxw 2l0f 2l0t 2l14 2l1l 2l29 2l2l 2l9s 2ld7 2leh 2lfw
2lkm 2lox 2lp0 2lp4 2lpb 2lqc 2lqh 2luh 2lvo 2lww 2lxm 2ly4
2lz6 2m04 2m0g 2m0j 2m5a 2m5b 2m86 2mbb 2mcn 2mej 2mfq 2mj5
2mlx 2mly 2mlz 2mnu 2mp0 2mre 2mro 2mur 2mv7 2mws 2mzd 2n01
2n1d 2n2h 2n8j 2nbv 2nqd 2o3b 2o8v 2omu 2omw 2omx 2oob 2ot3
2oul 2oza 2p43 2p44 2p45 2p47 2p48 2p49 2p8q 2pon 2ptc 2ptt
2px9 2qc1 2qna 2qur 2qxv 2rmk 2rms 2rnr 2roz 2rr3 2ru4 2ruk
2rvb 2sic 2sni 2tgp 2uuy 2uyz 2v3b 2v4z 2v52 2v6x 2v8s 2v9t
2vay 2vda 2vdb 2ver 2vln 2vlo 2vlp 2vlq 2vog 2voh 2voi 2vsm
2w84 2wd5 2wel 2wg4 2wh6 2wo2 2wo3 2wp3 2wpt 2wwk 2wy8 2x1x
2xgy 2xpx 2xtt 2y9m 2ygg 2z58 2z7f 3ajb 3alz 3aon 3au4 3beg
3bh6 3blh 3bn3 3bzd 3c4o 3c4p 3c59 3c5t 3ch5 3cqc 3cqg 3cx6
3cx7 3cx8 3d7t 3ddc 3di3 3doe 3dvm 3e1z 3f1p 3fhc 3fii 3fju
3fp6 3fpu 3gc3 3gj3 3gj6 3gni 3gqi 3gty 3gxu 3h3g 3h6g 3h8k
3hct 3idc 3iol 3ixe 3jza 3k1r 3k8p 3kj0 3kj1 3kj2 3knb 3kuc
3kud 3kv4 3kw5 3kyi 3l9j 3lbx 3lms 3m18 3m63 3mca 3me2 3mj7
3mzg 3mzw 3n00 3n06 3n0p 3n4i 3ncb 3ncc 3nvn 3o34 3o40 3o43
3o5t 3ohm 3oiq 3ojm 3oky 3ol2 3olm 3ona 3oun 3ouw 3oux 3p71
3p92 3p95 3pnr 3qc8 3qhy 3qq8 3qsk 3qwq 3rgf 3ro2 3sgb 3sjh
3sri 3t04 3tac 3tei 3tg1 3tgk 3tkl 3tnf 3tu3 3tz1 3u43 3u82
3u9z 3ujg 3ukw 3ukx 3ukz 3ul0 3ul1 3ul4 3uyo 3uyp 3uzq 3uzv
3v1c 3v6b 3v96 3vv2 3vyr 3w8h 3w8i 3wa5 3wdg 3wqb 3wwn 3zet
3zkq 3znz 3zo0 3zu7 3zwz 3zyi 4a49 4an7 4ap2 4apf 4apx 4aqe
4awx 4b1x 4b1y 4b93 4bd9 4bfi 4bkx 4bru 4brw 4c2a 4c4k 4c4p
```

4c5g 4c7n 4c9b 4cj0 4cj1 4cmm 4ct0 4cu4 4d0g 4d0n 4dbg 4did
4dj9 4ds8 4dt1 4dvg 4dxa 4dzu 4dzv 4ehq 4eig 4ekd 4etp 4euk
4exp 4f0a 4f38 4f48 4fza 4fzv 4g01 4g6u 4gaf 4gi3 4giq 4gn4
4h5s 4h6j 4hcn 4hcp 4hdo 4hep 4hff 4hrl 4i6l 4ika 4iop 4iu3
4iyp 4j2y 4j32 4je4 4jeg 4jeh 4jeu 4js0 4jw2 4k0a 4k5a 4ka2
4kr0 4krl 4ksd 4kt1 4kt3 4l0p 4l67 4lad 4lgr 4lzx 4m1l 4m5f
4mrt 4n7z 4nm3 4nqw 4nso 4nu1 4nzl 4nzw 4ol0 4p3y 4pas 4pbz
4per 4plo 4pou 4pqt 4pw9 4q5u 4qlp 4qxa 4rey 4rs1 4rt6 4rws
4tq1 4u32 4u4c 4u97 4udm 4uem 4uf1 4w6w 4w6x 4w6y 4wem 4wen
4wnd 4x33 4x7s 4xl5 4xwj 4xxb 4y5o 4y61 4yc7 4yeb 4yh7 4yj4
4yl8 4yn0 4yoc 4yvq 4yyp 4z9k 4zgm 4zgq 4zgy 4zii 4zk9 4zqu
5ajj 5b64 5b75 5b76 5b77 5b78 5bnq 5cxb 5cyk 5d1k 5d1l 5d1m
5d3i 5dc4 5dfw 5djt 5dob 5e6p 5e95 5ee5 5eg3 5elu 5eo9 5eql
5f4e 5f5s 5fr1 5fr2 5fzt 5g15 5g1x 5gjk 5glh 5gpg 5h3j 5h7y
5h9b 5hpk 5hps 5hu3 5hvf 5ij0 5ij9 5imk 5imm 5imt 5inb 5ixd
5j56 5j57 5j8h 5jds 5jjd 5jw7 5jw9 5k22 5k8q 5kve 5kxh 5ky0
5ky4 5ky5 5l21 5l8j 5lhn 5li1 5lxm 5lz3 5lz6 5m2j 5m72 5ma3
5ma4 5ma6 5me5 5ml9 5mtj 5mtm 5mtn 5mv8 5mv9 5my6 5npo 5nqf
5nqg 5nus 5nwm 5o2t 5o90 5oaq 5oen 5omn 5oy9 5oyl 5szh 5szi
5szj 5szk 5t0f 5tar 5tp6 5tvq 5tzp 5u4k 5u4m 5ua4 5ufe 5uk5
5un7 5uwc 5uzu 5v62 5v69 5v6a 5vkl 5vko 5vmo 5vwy 5vz4 5vzm
5wgg 5wos 5wpa 5wrv 5wuj 5xbf 5xeq 5xiu 5xln 5xoc 5xod 5xv8
5yi8 5yip 5yqz 5yr0 5ywr 5z2w 5zau 6aaf 6akm 6amb 6arq 6aw2
6azp 6b6u 6ba6 6bmt 6bw9 6cbp 6ch3 6d13 6d4p 6dgf 6dsl 6eg0
6er6 6f0f 6f2g 6f9s 6fbx 6fc3 6ff3 6fg8 6fp7 6fub 6fud 6fuz
6fv0 6g04 6gbg 6gd5 6gho 6gum 6gvl 6h16 6h46 6h47 6h9n 6har
6her 6hul 6i2m 6i3f 6idx 6ihb 6im9 6imf 6ird 6ire 6isc 6iu7
6iua 6ivu 6iw8 6iwa 6iwd 6j4o 6j4s 6jb2 6jb8 6jcs 6jjw 6jwj
6k06 6kbm 6kbr 6m7l 6mav 6mba 6mc9 6mud 6n85 6n9d 6ne1 6ne2
6ne4 6on9 6oqj 6oqk 6osw 6ov2 6pnp 6pnq 6qb3 6umt

List of pdb ids of protein-drug complexes:

10gs 1a30 1bcu 1e66 1f8b 1f8c 1f8d 1gpk 1h23 1hfs 1hnn 1igj
1jyq 1kel 1lbk 1lol 1loq 1lor 1mq6 1n1m 1n2v 1nvq 1o3f 1o5b
1os0 1oyt 1p1q 1ps3 1q8t 1q8u 1qi0 1r5y 1sln 1sqa 1u1b 1u33
1uto 1vso 1w3k 1w3l 1w4o 1xd0 1yc1 1z95 1zea 2brb 2cbj 2cet
2d1o 2d3u 2fvd 2g70 2gss 2hb1 2iwx 2j62 2j78 2jdm 2jdu 2jdy
2obf 2ole 2p4y 2pcp 2pq9 2qbp 2qbr 2qft 2qmj 2r23 2v00 2vl4
2vo5 2vot 2vvn 2vw5 2w66 2wbg 2wca 2weg 2wtv 2x00 2x0y 2x8z
2x97 2xb8 2xhm 2xnb 2xy9 2xys 2yfe 2yge 2yki 2ymd 2zcq 2zcr
2zjw 2zwz 2zx6 2zxd 3acw 3ag9 3ao4 3b3s 3b3w 3b68 3bfu 3bkk
3bpc 3cft 3cj2 3coy 3cyx 3d4z 3dd0 3dxg 3e93 3ebp 3ehy 3ejr
3f17 3f3a 3f3c 3f3e 3f80 3fcq 3fk1 3fv1 3g0w 3g2n 3g2z 3gbb
3gcs 3ge7 3gnw 3gy4 3huc 3i3b 3imc 3ivg 3jvs 3k5v 3kgp 3kv2
3kwa 3l3n 3l4u 3l4w 3l7b 3lka 3mfv 3mss 3muz 3myg 3n7a 3n86
3nox 3nq3 3nw9 3oe5 3owj 3ozt 3pe2 3pww 3pxf 3s8o 3su2 3su3
3su5 3u9q 3udh 3ueu 3uex 3uo4 3uri 3utu 3vd4 3vh9 3zso 3zsx
4de1 4de2 4des 4dew 4djr 4djv 4g8m 4gid 4gqq 4tmn
